# Rachel Challice's Vow

**Published:** 1888-01-21

**Authors:** Lina Nevill

**Affiliations:** Author of "A Future on Trust," "Juno," &c.


### RACHEL CHALLICE'S YOW.

By LINA NEVILL, Author of "A Future on Trust," "Juno," &c.

CHAPTER III.—(*continued.*)

MR. STEPHENS and his son were sitting over their dinner the day before that fixed for Mrs. Challice's funeral.

"I trust that Mr. Jones will have the good feeling to bury her free. She is a poor lone widow woman, as has kept herself above the parish all these years," observed the elder man, spearing up a potato on the end of his knife and carrying it to his mouth as he spoke.

"It sickens me, it do, to see the troubles and trials of this world turned into an occasion for profit," he concluded, as soon as Sutton's Magnum Bonum permitted him renewed power of speech.

This theory was hard on the undertakers, the doctors, the lawyers, and many other trades, whose profit and very subsistence depend wholly or in part on the ills and adversities to which their neighbours are subject. If there were to be a jubilee year's exemption from sickness and mortality, the run on the Bankruptcy Court during the twelve months' reprieve would be alarming.

"I shall go to the funeral, father," observed David with a half-assumed air of indifference.

"There's no call for you to do that," replied his father sharply.

"Only it would look friendly, and show a bit of respect for the poor old woman."

"If it's merely a question of that, David, and them is your feelings, I'll follow the funeral myself. I'm by many years nearer in age to the old lady who's gone than you are. Folks might say it's the living and not the dead you was a-following of."

The old man peered curiously at his son with his little, ferrety eyes, and speared up another potato, this time from the dish in front of him.

David made no reply to the insinuation.

"So that is settled, my lad," said Mr. Stephens heartily. "You'll keep an eye on the mill to-morrow, while I pay my last respects to her who's gone. I always held a great respect for old Mrs. Challice myself; I always do for them as strive to keep themselves above parish relief. And if I go, I'll drop Mr. Jones a hint about the money; though he's that pigheaded I make no doubt he'll take the subject unpleasantly."

Mr. Stephens laid down his knife and fork, finished his glass of beer, and pushed back his chair, to signify that the discussion and the meal, so far as he himself was concerned, was ended.

David looked up slowly.

"It's very good of you to promise to go to the funeral, father, and I am sure the girls will be pleased at the respect shown their grandmother; but I think, for all that, I'll go myself too. James can see to the mill well enough for the hour or two we shall both be away."

Mr. Stephens eyed his son keenly.

"See here, my lad," he said deliberately, "I fancy I know your feelings. You don't go to see the old woman put in her grave, but to have a sight of and a word with Esther. I've spoke my mind on the subject before, so there's no call to do so again. Marry the girl if you will. I can't prevent it, and you are no longer an infant in the eyes of the law. But the very day you do so I make a new will, and leave everything to go towards covering the burying expenses annually of as many deserving inhabitants of Aberclwyn, free of charge, as far as the money will go. That is what I shall do, David; and have my name and my bequest printed up in gold letters on a black board by the church door. Besides, Esther isn't to my taste. If you had to choose a Challice for a wife, I'd rather you took the other one."

"Rachel!" exclaimed David in astonishment. "Why, you might as well try to make love to a millstone—you'd get as much life out of it as from her."

"I don't think so," replied the old man, rising from his chair, and putting on the soft, flour-besprinkled hat he habitually wore in the mill.

David stared after him in amazement, unable to master his surprise. His father's words revealed Rachel to him in a new and never before dreamed-of light.

David's was a weak character at bottom, easily led and influenced by stronger natures than his own; and to his father's judgment—though he would not have owned it—he secretly deferred in all things. But, as in most weak natures, he had a strong vein of obstinacy in his composition. And though he began to think he had possibly misjudged Rachel, he still held doggedly to his first belief that Esther was the wife for him.

"I can't understand his meaning," he found himself often reflecting that afternoon, as the big mill-wheel turned the machinery, and crushed out the white heart from the brown corn husks, sending the dust flying in a thick white fog throughout the building.

"The old man never says a thing without a reason," he concluded; "but I'll marry Esther, and not Rachel, if I can get him by hook or by crook to consent to it. I do think it hard lines not to be able to please oneself where a wife is concerned. But what did he mean when he said Rachel was his choice? I'll take a bit more pains to talk to her next time I see her, and find out for my own satisfaction whether she is or is not the sort of girl I and everyone else but father takes her for. But Esther is the girl for my money."

Then he paused in his soliloquy, pulled up by the thought of how very little of that desirable article he would possess should he persist in making Esther Challice his wife.

"Bury a dozen old folks free every year!" he said to himhimself, grimly, "and let his only son starve! I reckon it would be a deal better if he left the dead to the parish, and provided for the living. Charity begins at home—so they say; but not as regards you, David, my boy. You must wait till you are dead to profit by your father's money. And then you and your wife, according to the gilt rules on the blackboard, can be buried for nothing out of the charitable bequest of your own father, provided you are deserving objects for that said charity! It sounds queer, and no mistake."

The big mill-wheel went round and round, and the problem as to the superiority of the two sisters one above the other revolved as persistently in David's head.

But at the end of the day, when the wheel was stopped, the machinery at rest, and the white dust was settling in thick layers over everything inside the mill, David found himself no nearer to the solution of the puzzling idea raised by his father's words. And when he went to bed that night, he took the unsolved problem upstairs with him.

CHAPTER IV.—RACHEL'S RESOLVE.

"——— As the bloom fades from the face of nature, so the gloss
Of his warm feelings faded with their freshness."

A YEAR had gone by since old Mrs. Challice died. Rachel and Esther Challice stood together in the cottage garden. The year had passed quietly and evenly with them. They kept on the old home, just managing to live on the tiny pension allowed them by Lord Merthyn, who said he could not let old John Challice's grandchildren be left to starve.

Esther looked the same as ever. People who feel little change little; time touches them lightly, leaving few traces of his transit. It was Rachel who had altered. The face that before had looked so immovable had gained a play of expression that had never been there in the old days of self-restraint and repression. The grey eyes beneath their long black lashes had kindled into life, and had lost the dull fixed look that they had hitherto invariably worn.

If a shadow of discontent rested on the face of either of the sisters, it was on Esther's, not on Rachel's. A wonderful calm gladness lit up now the elder girl's features, and made the pale oval face beautiful.

Esther's fretful voice broke the silence.

"I call it hard, Rachel, very hard; but it's just like you. You never think of, or feel for anyone but yourself. Poor dear Granny always said so, and you know as well as I do that it is true."

Things had gone crossways with Esther lately, which accounted for her fretful tone and angry accusation.

Lovers were a necessity to her shallow nature; she never could be happy unless she were a centre of attraction and admiration, and lovers were a very scarce article just at present with Miss Esther Challice. She had but one *boná fide* lover on her list, and concerning him—David Stephens—she was at that moment complaining to her sister in her fretful, spoilt-child voice.

"I do call it hard," she continued. "David cared for me first. He never so much as looked at you till you laid yourself out to take him away from me. And now you know he talks to you every bit as much as he does to me. I call it very selfish and mean and unfair of you, Rachel, that I do!"

The crimson blush that had risen to Rachel's cheeks at mention of David's name faded, leaving them pale as usual.

Poor Esther's accusation was partly true. She was no longer the one woman in the world to David Stephens.

Rachel, with a woman's rarely erring instinct in such matters, knew that the young man regarded her with an interest she had formerly failed to win from him.

Pondering over his father's words, David had set himself to prove their truth. The trial begun as an experiment developed into sober earnest. It was such a new experience to see the pale, cold features kindle into life and animation as he talked to Rachel, to see a smile part the firm, closed lips. The experiment grew beyond an interest, it became a pleasure; and David's greatest delight now was to see his Galatea thaw, unbend, rouse into life, and finally come down from her marble pedestal, and advance shyly to meet him.

But till her sister's words revealed the truth, Rachel had never dreamed of supplanting Esther in David's affections. If this state of affairs was not mere imagination it must be checked at once. Had not Rachel vowed to protect her sister's welfare at any cost to herself?

The difficulty would be to find out how the land really lay. It would be awkward to assume an attraction where none might exist, and place her—Rachel—in a false and very uncomfortable position; yet to let matters drift might be even more fatal to Esther's chances.

"It is very cruel of you, Rachel, and I'm so unhappy, I wish I were dead like Granny, and then perhaps you might be sorry, and wish you hadn't been so mean!"

Esther's pretty baby face was puckered, and a few tears were running down her plump, peach-like cheeks.

It was by no means an unusual occurrence to see the younger Miss Challice cry; but it was a sight that, accustomed to it as she was, always filled Rachel with compunction.

"I couldn't live if David got not to care for me," she whimpered. "I never loved anyone but him. If he got tired of me because someone else laid herself out to take him away, I should go and drown myself straight. You never loved anyone in all your life, Rachel, so you can't imagine for a moment how wretched I feel."

It was all very ludicrous and high flown. A stranger or impartial observer would have laughed to hear that pretty little soft plump girl talk about drowning herself. One could quite as easily imagine a fluffy white kitten calmly walking to the margin of the river and deliberately precipitating herself into the cold, uninviting water.

But where Esther was concerned, Rachel's usual common sense deserted her.

She put her arm round the child, and tried to check the petulant noisy sobbing.

"Don't cry, dear. David loves you, I am sure. It is all nonsense about his caring for me."

The sobs ceased instantly.

"You are quite certain, Rachel? Well, I suppose I must believe you. And it did seem odd to me, too," she added, with a little self-satisfied laugh. "You know, Rachel, it is always I who have admirers, not you."

"Of course, dear," replied her sister. But there was a hard metallic ring in her voice; more of the cold, constrained tones of former years than Esther had heard for many months.

"And you won't talk to David, or let him talk to you, and sit by you as he does now?" pursued Esther, eagerly. "He won't do it unless you encourage him," she added, jealously.

"No, we will not talk to, nor sit by each other."

A new resolve had suddenly flashed into the elder girl's mind. A course of action had been revealed to her by which her vow could be kept to the letter, and the road left unmolested to Esther.

"You won't talk to him any more, Rachel?" Esther reiterated, anxiously. This return of her sister's former cold manner puzzled and frightened her.

"Only to-night, Esther; David is coming to-night, and I must have one more talk with him alone. Don't be frightened, it shall be our last."

"How do you know he is coming to-night?" asked Esther, her suspicions newly aroused by this knowledge on Rachel's part.

"He told me so when I met him in Aberclwyn this morning. I thought you heard him say so, or else I would have mentioned it."

"But if I do leave you alone, how can I tell what you will say to him!" burst out Esther with renewed fretfulness and mistrust. "You are so different from other girls. There is no telling what you may say or do. You have been so much nicer lately, I hoped it was going to last; but to-night you are just as cold and disagreeable as ever. I really think poor dear Granny was right in all she used to say about you."

Rachel took no notice of the unkind words. Indeed, she scarcely heard them.

"What do you want to say to David?" repeated Esther.

She was one of those tactless people who never let an unpleasant subject drop, but who harp for hours on the same disagreeable topic.

"You shall know some day; not just now."

Rachel had caught sight of a man's figure coming along the road; her quick eyes detected in a moment who it was, and the hot blood rushed to her cheeks.

She wanted to meet him naturally and see him alone without any fuss or parade of sending Esther away, and making a point of a private interview; but Esther was difficult to manage. She could be led, but not driven.

"Run in and make tea, dear," she said hurriedly. "David shall see what a good little housekeeper you are. I'll go and meet him, and bring him in presently for us all to have tea together.

Esther demurred, raised objections, and at length unwillingly complied.

Then Rachel was free.

With a calm, deliberate air she walked down the garden path, opened the gate, and stepped out into the road, meeting David face to face.

"Let us walk on a little way," she said in her usual even tone of voice. "Esther is getting tea, and does not want us directly. Come a little way up the mountain, and see the sun set on the other side."

David was glad enough of the excuse. Esther did not often let him and Rachel enjoy a *tête-à-tête*, and Rachel was always more stiff and unbending in the presence of a third person. Just now, too, the star of the elder sister was in the ascendant. Esther had been silly and reproachful last time David had walked with her; and he was beginning to think that after all his father was right, and Rachel would make the better wife in the long run.

So he willingly acceded to her proposal, and the two began to climb the steep green hill that sloped down to the very verge of the road.

It was a stiff climb, and neither spoke much till they reached the top.

Then Rachel seated herself on a grey boulder, and pointed to another close by.

"Sit there, David," she said, "and let us have a talk."

And with the after-glow shining full on her pale face; with the lovely valley with its glittering lakes and tumbling rivers spread out at their feet; with only the flocks of mountain sheep and the sure-footed wild goats as audience, Rachel with a steady voice began rehearsing the part she had learned.

*(To be continued.)*

